# Cannabinoid receptor-2 attenuates neuroinflammation by promoting autophagy-mediated degradation of the NLRP3 inflammasome post spinal cord injury

**DOI:** 10.3389/fimmu.2022.993168

**Published:** 2022-09-26

**Authors:** Fan Jiang, Mingjie Xia, Yanan Zhang, Jie Chang, Jiang Cao, Zhongkai Zhang, Zhanyang Qian, Lei Yang

**Affiliations:** ^1^ Department of Orthopedics, Taizhou People’s Hospital, Nanjing Medical University, Taizhou, China; ^2^ Department of Orthopedics, Nanjing First Hospital, Nanjing Medical University, Nanjing, China; ^3^ Postgraduate School, Dalian Medical University, Dalian, China; ^4^ Department of Orthopedics, Affiliated First Hospital of Nanjing Medical University, Nanjing, China; ^5^ Department of Orthopedics, Shandong Provincial Hospital Affiliated to Shandong First Medical University, Jinan, China; ^6^ Department of Orthopedics, Zhongda Hospital, Nanjing, China; ^7^ School of Bioinformatics Engineering, Nanjing Medical University, Nanjing, China

**Keywords:** cannabinoid receptor-2, neuroinflammation, NLRP3, autophagy, spinal cord injury

## Abstract

**Background:**

Neuroinflammation following spinal cord injury (SCI) results in prolonged neurological damage and locomotor dysfunction. Polarization of microglia is vital to regulation of neuroinflammation, although the underlying mechanisms have not yet been elucidated. Endocannabinoid receptor subtype 2 (CB2R) is reported to ameliorate neurodegeneration *via* immunomodulation activities. However, the underlying machinery in the context of SCI remains unclear.

**Methods:**

A lipopolysaccharide-induced microglia inflammation model and a mouse model of SCI were employed to investigate the regulatory role of CB2R in the polarization of microglia in response to excess neuroinflammation. Markers of inflammation and autophagy were measured by Western blot analysis, immunofluorescence, flow cytometry, and enzyme-linked immunosorbent assays. Histological staining with hematoxylin and eosin, Nissl, and Luxol^®^ fast blue was conducted using commercial kits. The locomotor function of the hindlimbs of the experimental mice was evaluated with the Basso Mouse Scale, Louisville Swim Scale, and footprint assay.

**Results:**

The results showed that CB2R promoted M2 differentiation, increased interleukin (IL)-10 expression, and inhibited M1 differentiation with decreased expression of IL-1β and IL-6. CB2R activation also increased ubiquitination of the NLRP3 inflammasome and interacted with the autophagy-related proteins p62 and microtubule-associated proteins 1B light chain 3. Treatment with the CB2R activator JWH-133 reduced loss of myelin, apoptosis of neurons, and glial scarring, leading to improved functional recovery of the hindlimbs, while the CB2R antagonist AM630 produced opposite results.

**Conclusion:**

Taken together, these results suggested that CB2R activation attenuated neuroinflammation targeting microglial polarization by promoting NLRP3 clearance, thereby facilitating functional recovery post-SCI.

## Introduction

Spinal cord injury (SCI) is a deteriorative neuropathy that results in irreversible neurological dysfunction and severe physio-psychological damage to the patient (1, 2). However, the pathogenesis of SCI remains unclear, as available reports have mostly focused on either the induction of neuroinflammation, accumulation of reactive oxygen species (ROS), or development of neurotoxicity (3). Microglia are indispensable components of pathological events (4). Damage to the spinal cord initiates various inflammatory responses *via* immunoregulation of microglial injury and subsequent activation of damage-associated molecular patterns, which promote activation of microglia in the resting state *via* multiple molecular signaling pathways, leading to increased differentiation of M1 microglia that release destructive pro-inflammatory mediators and induce ROS generation and a lack of M2 microglia, thereby limiting excess inflammation, but fueling neuroinflammation (5–7). The cross-kingdom efficacy of microglial polarization as an immune-inflammatory junction suggests a potential target as a defensive mechanism, although the specific components remain unclear.

The NLRP3 (NOD-, LRR-, and pyrin domain-containing 3) inflammasome, an intracellular signaling multiprotein complex, is reported to be activated in M1 microglia. It promotes production of numerous inflammatory amplifiers and has been implicated in the pathogenesis of various non-infectious diseases (8–10). As the most characteristic inflammatory sensor molecule, NLRP3 triggers activation of capase-1 and maturation of interleukin (IL)-1β and IL-18 (11, 12). Moreover, autophagy is a scavenging mechanism that delivers damaged organelles as well as excess proteins to lysosomes. Multiple lines of evidence show that accumulation of the NLRP3 inflammasome induces autophagosome formation, which limits inflammasome activity (13–15). However, the therapeutic effect of the destruction of the NLRP3 inflammasome *via* regulation of autophagy to alleviate secondary injury post-SCI remains unclear.

Previous studies have suggested that activated microglia produce endocannabinoids and express cannabinoid receptor subtypes that suppress neuroinflammation (16). In addition, endocannabinoids were reported to be an effective adjuvant to inhibit activation of NLRP3 inflammasome (17). The endocannabinoid system is mainly composed of endocannabinoid receptor subtype 1 (CB1R), endocannabinoid receptor subtype 2 (CB2R), and endocannabinoids like 2-arachidonoyl glycerol, anandamide, and other structurally related lipids, as well as related enzymes for transportation, synthesis, and degradation, which are involved in a variety of physiological functions and pathological processes (18–20). Of interest, activation of CB2R in the central nervous system plays a protective role against neurodegenerative diseases attributed to immune regulation (21–25). Previous reports suggest that targeting CB2R manipulates M1/M2 transition of macrophages in response to inflammation and regulates autophagy-related signaling (26, 27). However, the unique mechanisms underlying regulation of neuroinflammation post-SCI *via* CB2R and autophagy remain elusive.

In the present study, a model of lipopolysaccharide (LPS)-induced inflammation of microglia and a mouse model of contusion-induced SCI were employed to explore the role of CB2R in SCI-induced neuroinflammation *in vitro* and *in vivo*, respectively. The results showed that activation of CB2R facilitated phenotype transition of microglia to the M2 state, rather than M1, restrained neuronal apoptosis, and protected neurological functions, suggesting that CB2R attenuated SCI-induced neuroinflammation by promoting ubiquitination and clearance of NLRP3 through autophagy in microglia.

## Materials and methods

### Study approval

The animal study protocol was approved by the Institutional Animal Care and Use Committee of Nanjing Medical University (Nanjing, China) and conducted in accordance with the guidelines described in “Animal Research: Reporting of *In Vivo* Experiments”.

### Antibodies

The antibodies (Abs) mentioned in this study are listed in **Table 1**.

**Table 1:** The information of Abs

Antibody Name #Cat.	Source	Species	Application	Dilution rate
**CNR2 Recombinant Rabbit Monoclonal Antibody # 703485**	Invitrogen	Rb	WB/IF	1:500
**NLRP3 Rabbit mAb #49012**	Signalway Antibody	Rb	WB/IF/IP	1:1,000/1:200/1:30
**Phospho-AMPKα (Thr172) (D4D6D) Rabbit mAb #50081**	CST	Rb	WB	1:1,000
**AMPKα (D63G4) Rabbit mAb #5832**	CST	Rb	WB	1:1,000
**Phospho-ULK1 (Ser757) (D7O6U) Rabbit mAb #14202**	CST	Rb	WB	1:1,000
**ULK1 (D8H5) Rabbit mAb #8054**	CST	Rb	WB	1:1,000
**LC3A Mouse Monoclonal Antibody(5G10) #44176**	Signalway Antibody	Ms	WB	1:1,000
**Anti-Ubiquitin (linkage-specific K48) antibody [EP8589] #ab140601**	Abcam	Rb	WB	1:1,000
**LC3B Polyclonal Antibody #29075**	Signalway Antibody	Rb	WB/IF	1:1,000/1:100
**Phospho-NF-κB p65 (Ser536) (93H1) Rabbit mAb #3033**	CST	Rb	WB/IF	1:1,000
**NF-κB p65 (D14E12) XP^®^ Rabbit mAb #8242**	CST	Rb	WB/IF	1:1,000/1:500
**SQSTM1/p62 (D6M5X) Rabbit mAb (Rodent Specific) #23214**	CST	Rb	WB	1:1,000
**HRP-conjugated GAPDH Monoclonal antibody # HRP-60004**	Proteintech	Ms	WB	1:10,000
**Goat Anti-Rabbit IgG Secondary antibody (H+L), HRP #YFSA02**	YIFEIXUE BioTech	Goat	WB	1:10,000
**Goat Anti-Mouse IgG Secondary antibody (H+L), HRP # YFSA01**	YIFEIXUE BioTech	Goat	WB	1:10,000
**Anti-iNOS antibody #ab15323**	Abcam	Rb	IF	1:100
**Arginase-1 (D4E3M™) XP^®^ Rabbit mAb #93668**	CST	Rb	IF	1:50
**Anti-Iba1 antibody [EPR16588] #ab178846**	Abcam	Rb	IF	1:500
**GFAP (GA5) Mouse mAb #3670**	CST	Ms	IF	1:600
**Neurofilament-H (RMdO 20) Mouse mAb #2836**	CST	Ms	IF	1:400
**Myelin Basic Protein (D8X4Q) XP^®^ Rabbit mAb #78896**	CST	Ms	IF	1:50
**Anti-NeuN Antibody, clone A60 #MAB377**	Millipore	Ms	IF	1:100
**Anti-Annexin V/ANXA5 Antibody [EPR3980] #ab108194**	Abcam	Rb	IF	1/50
**Alexa Fluor^®^ 594 AffiniPure Fab Fragment Goat Anti-Rabbit IgG (H+L) #111587003**	Jackson ImmunoResearch	Goat	IF	1:500
**Alexa Fluor^®^ 488 AffiniPure Fab Fragment Goat Anti-Rabbit IgG (H+L) #111547003**	Jackson ImmunoResearch	Goat	IF	1:500
**Alexa Fluor^®^ 594 AffiniPure F(ab’)₂ Fragment Goat Anti-Mouse IgG (H+L) #115586003**	Jackson ImmunoResearch	Goat	IF	1:500
**Alexa Fluor^®^ 488 AffiniPure F(ab’)₂ Fragment Goat Anti-Mouse IgG (H+L) #115546003**	Jackson ImmunoResearch	Goat	IF	1:500
**BD Pharmingen™ PE Rat Anti-Mouse F4/80 #565410**	BD Biosciences	Rat	FCA	1:500
**BD Transduction Laboratories™ FITC Mouse Anti-iNOS/NOS Type II #610330**	BD Biosciences	Ms	FCA	1:500
**CD206 (MMR) Monoclonal Antibody (MR6F3), APC, eBioscience™#17-2061-82**	Invitrogen	Rat	FCA	1:500

### Cell culture

BV2 microglia and human embryonic kidney (HEK)-293T cells were obtained from the Institute of Cell Research, Chinese Academy of Medical Sciences (Shanghai, China) and cultured in Dulbecco’s modified Eagle medium (KGM12800-500; Nanjing KeyGen Biotech. Co. Ltd., Nanjing, China) supplemented with 10% fetal bovine serum (Gibco, Grand Island, NY, USA). At 80% confluence, the microglia and HEK-293T cells were pretreated for 24 h with the CB2R activator JWH-133 (10 nM; B7941; APExBIO Technology LLC, Houston, TX, USA) or antagonist AM630 (40 nM; A3168; APExBIO Technology LLC) dissolved in 0.1% dimethyl sulfoxide (KGT5131; Nanjing KeyGen Biotech. Co. Ltd.). Inflammation was stimulated by treatment with 1 μg/ml of LPS (Sigma-Aldrich, St. Louis, MO, USA) for 24 h.

### Establishment of a mouse model of SCI

Male C57BL/6J mice, aged 8 weeks, were obtained from the Experimental Animal Center of Nanjing Medical University and randomly assigned to one of the following four groups: sham group (laminectomy and daily intraperitoneal [i.p.] administration of a vehicle solution composed of 5% ethanol, 5% dimethyl sulfoxide, 5% Tween 20, and 85% normal saline), SCI group (SCI and i.p. administration of the vehicle solution), SCI+J group (SCI and i.p. administration of JWH-133 at 2 mg/kg per day), or SCI+A group (SCI and i.p. administration of AM630 at 2 mg/kg per day). JWH-133 and AM630 were initially administered 30 min prior to SCI and continued for 3 days. For SCI modeling, mice were anesthetized with ketamine (80 mg/kg) and xylazine (4 mg/kg) prior to laminectomy. A moderate contusion to the spinal cord at T10 was created with a 5-g impactor dropped from a height of 5 cm (28). The bladder of all mice with SCI was manually emptied once per day until the development of incontinence.

### Western blot analysis

Proteins were extracted from cells and tissues using a total protein extraction kit (KGP2100; Nanjing KeyGen Biotech. Co. Ltd.) in accordance with the manufacturer’s protocol. Following quantification using an enhanced bicinchoninic acid kit (P0010; Beyotime Institute of Biotechnology, Shanghai, China), equal amounts of protein from each group were separated by sodium dodecyl sulfate polyacrylamide gel electrophoresis and then transferred to polyvinylidene fluoride membranes (thickness, 0.45 μm; EMD Millipore Corporation, Billerica, MA, USA), which were blocked with 5% skim milk diluted in tris-buffered saline-Tween-20 for 1 h at room temperature and then incubated with primary Abs overnight at 4°C, followed by secondary Abs for 1 h at room temperature. Afterward, the membrane was visualized using a chemiluminescence system (4600; Tanon Science and Technology Co., Ltd., Shanghai, China) and analyzed with ImageJ software (https://imagej.nih.gov/ij/).

### Enzyme-linked immunosorbent assay

The supernatant of the cell culture of each group was collected by centrifugation at 3,000 rpm for 10 min and stored at −80°C. Expression levels of inflammatory markers (IL-1β, IL-6, and IL-10) were measured using appropriate ELISA kits [EK201B, EK206, EK210; Multisciences (Lianke) Biotech Co., Ltd., Hangzhou, China] in accordance with the manufacturer’s protocols. Absorbance was determined at 550 nm using a multifunctional microplate reader (Synergy™ HT; BioTek Instruments, Winooski, VT, USA).

### Flow cytometry

Polarization of microglia was induced by incubation with phycoerythrin (PE)-conjugated F4/80 Ab plus fluorescein isothiocyanate-conjugated Ab against inducible nitric oxide synthase (iNOS) or PE-conjugated F4/80 Ab plus allophycocyanin-conjugated Ab against CD206 at 4°C for 30 min. Then, the proportions of polarized microglia in each group were determined using a BD FACSVerse™ flow cytometer (BD Biosciences, Franklin Lakes, NJ, USA). The generated dataset was analyzed using FlowJo software (version 8.0; https://www.flowjo.com/).

### Terminal-deoxynucleotidyl transferase mediated nick end labeling assay

Cell death in injured spinal cord tissue was determined using a one-step terminal-deoxynucleotidyl transferase mediated nick end labeling (TUNEL) apoptosis kit (KGA7062; Nanjing KeyGen Biotech. Co. Ltd.) in accordance with the manufacturer’s instructions. Briefly, tissue sections were incubated with a working buffer containing proteinase K at 37°C for 30 min, then washed three times with PBS for 5 min and incubated in DNase I solution at 37°C for 30 min. Afterward, the tissue sections were probed with an enzyme working buffer at 37°C for 1 h, then labeled with tetramethylrhodamine-conjugated streptavidin in the dark at 37°C for 30 min. The nuclei were counterstained with Fluoromount-G^®^, a water-soluble compound containing 4´,6-diamidino-2-phenylindole (0100; SouthernBiotech, Birmingham, AL, USA). The tissue sections were imaged with a fluorescence microscope (BX51; Olympus Corporation, Tokyo, Japan).

### Immunoprecipitation

BV2 and HEK-293T cells were lysed with protein lysis buffer on ice for 30 min and centrifugated at 12,000 × *g* and 4°C for 10 min. Then, 50 μl of the supernatant containing protein was collected as the “input” group, while the remaining supernatant was probed with Protein G Sepharose^®^ 4 Fast Flow beads (GE Healthcare Sverige AB, Stockholm, Sweden) coated with an Ab against NLRP3 at 60 rpm and 4°C overnight using a rotary table. The beads were boiled twice in 2× loading buffer at 100°C for 5 min and then washed at 120 rpm and 4°C.

### Immunofluorescence staining

Microglia were deparaffinated, rehydrated, and fixed with 4% paraformaldehyde (P0099; Beyotime Institute of Biotechnology) for 15 min for antigen retrieval and blocked using Immunol Staining Blocking Buffer (P0102; Beyotime Institute of Biotechnology) at room temperature for 1 h. The samples were probed with primary Abs overnight at 4°C, followed by secondary fluorescent Abs at room temperature for 1 h. The nuclei were counterstained with Fluoromount-G^®^ and the samples were observed using a fluorescence microscope.

### Histological staining

Histological staining with hematoxylin and eosin (H&E), Nissl, and Luxol^®^ fast blue (LFB) was conducted using commercial kits (G1120, G1436, G3240; Beijing Solarbio Science and Technology Co., Ltd., Beijing, China) in accordance with the manufacturer’s protocols. Sections were mounted with neutral balsam (G8590; Beijing Solarbio Science and Technology Co., Ltd.) and then photographed using a fluorescence microscope.

### Behavioral assessment

Locomotor function of the hindlimbs of the experimental mice was evaluated by two blinded researchers in accordance with the Basso Mouse Scale, the Louisville Swim Scale, and a footprint assay as previously described (29, 30).

### Statistical analysis

Statistical analysis was performed with Prism 8.3.0 software (GraphPad Software, Inc., San Diego, CA, USA) and the data are presented as the mean ± standard deviation. Comparisons between two groups were conducted using the unpaired *t*-test and among three or more groups using one-way or two-way analysis of variance followed by Tukey’s *post hoc* test. A probability (*p*) value < 0.05 was considered statistically significant.

## Results

### Expression patterns of CB2R in cord tissue post-SCI

**Figure 1:**
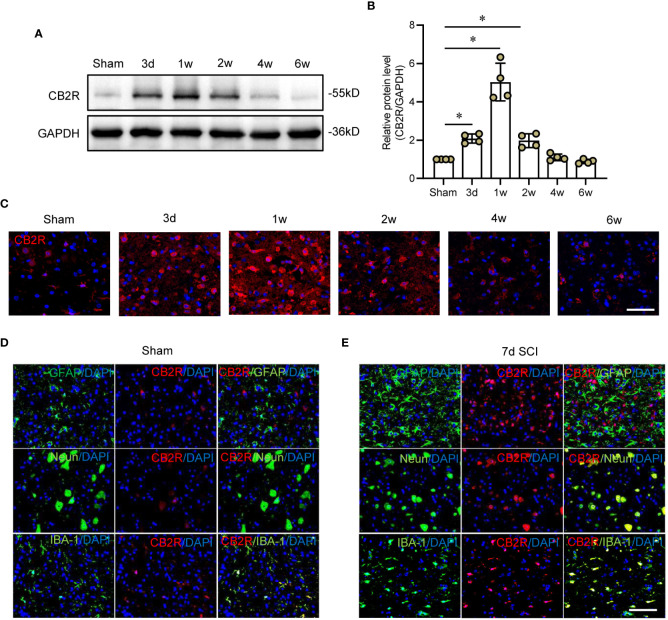
Expression patterns of CB2R in cord tissues post-SCI. **(A)** Representative protein band of CB2R on day 3 and weeks 1, 2, 4, and 6 post-SCI. **(B)** Quantitative analysis showing that CB2R expression was significantly increased on day 3 and weeks 1 and 2 post-SCI. **(C)** IF staining showing that CB2R (red) in cords of mice within 6W post-SCI. **(D)** IF staining of CB2R (red) with GFAP, Neun, or IBA-1 (green) in cords of sham mice. **(E)** IF staining of CB2R (red) with GFAP, Neun, or IBA-1 (green) in SCI mice on day 7 post-SCI. Scale bar = 100 μm. ^*^ *p* < 0.05.

CB2R protein expression was measured from day 3 to week 6 post-SCI (**Figure 1A**). Post-SCI, CB2R protein expression significantly increased and peaked by 1 week, then gradually decreased to week 6 (**Figure 1B**), which was consistent with the IF staining results (**Figure 1C**). Furthermore, IF staining on day 7 post-SCI showed that the distribution of CB2R expression largely overlapped with the neuron-specific marker NeuN and microglia-specific marker IBA-1 in both the uninjured and injured mice. In contrast, astrocytes positive for expression of glial fibrillary acidic protein (GFAP) had expressed relatively low levels of CB2R (**Figures 1D, E**). Hence, microglia were selected for further studies.

### CB2R regulates inflammation-mediated polarization of microglia

**Figure 2:**
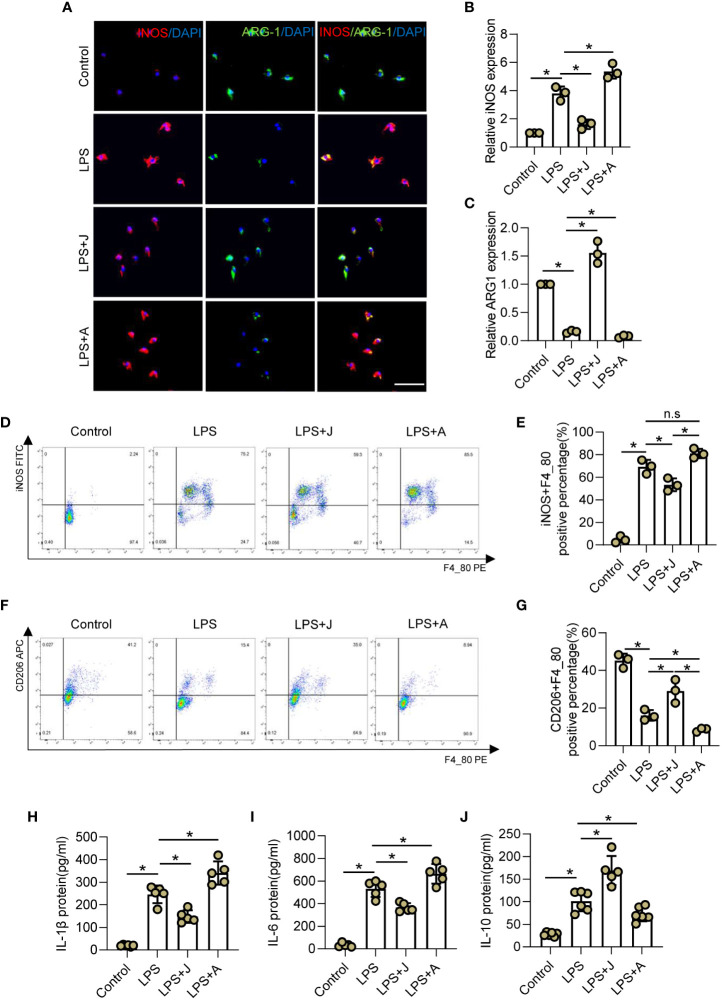
CB2R regulates inflammation-mediated polarization of microglia. **(A)** IF staining of iNOS (red) and ARG-1 (green) in BV2 microglia after LPS insult for 24 h with CB2R activation by JWH-133 (J) or inactivation by AM630 **(A)**. Scale bar = 50 μm. **(B)** Quantitative analysis indicating that CB2R significantly altered expression of the M1 marker iNOS during inflammation. **(C)** Quantitative analysis indicating that CB2R significantly altered expression of the M2 marker ARG-1 after LPS stimulation. **(D)** Flow cytometry results showing the relative proportion of M1 microglia (red) after LPS insult with and without CB2R activation or inactivation. **(E)** Quantitative analysis of M1 microglia. **(F)** Flow cytometry results showing the relative proportion of M2 microglia (red) after LPS insult with and without CB2R activation or inactivation. **(G)** Quantitative analysis of M2 microglia. ELISA results of IL-1β **(H)**, IL-6 **(I)**, and IL-10 **(J)** in the control, LPS, LPS+J (JWH-133), and LPS+A (AM630) groups. ^*^ *p* < 0.05. n.s means no significance.

The CB2R activator JWH-133 and antagonist AM630 were employed to determine whether CB2R participates in regulation of the immunophenotype of LPS-treated microglia (31, 32). Specifically, the expression patterns of the M1 subtype marker iNOS and M2 subtype marker ARG-1 were determined by IF staining (**Figure 2A**). The results showed that LPS markedly increased expression of iNOS, but reduced that of ARG-1 in microglia. Subsequent treatment with JWH-133 decreased LPS-stimulated upregulated expression of iNOS, but rescued pull-down of ARG-1, while treatment with AM630 aggravated LPS-stimulated upregulation of iNOS and inhibited expression of ARG-1 (**Figures 2B, C**). In addition, the proportion of polarized cells was determined by flow cytometry. The results showed that the proportion of iNOS+ M1 microglia was significantly increased after LPS stimulation, and activation of CB2R by JWH-133 remarkably reduced such increase, while inhibition of CB2R by AM630 further intensified this effect (**Figures 2D, E**). In contrast, LPS stimulation remarkably reduced the proportion of CD206+ M2 microglia, which was further decreased by treatment with AM630 but increased by treatment with JWH-133 (**Figures 2F, G**). LPS stimulation increased expression of IL-1β, IL-6, and IL-10, whereas JWH-133 decreased expression of IL-1β and IL-6, but increased that of IL-10. In contrast, AM630 increased expression of IL-1β and IL-6, but decreased that of IL-10 (**Figures 2H–J**).

### CB2R promoted ubiquitination and autophagy-induced degradation of NLRP3 *via* the adenosine monophosphate-activated protein kinase/unc-51-like autophagy activating kinase signaling pathway

**Figure 3:**
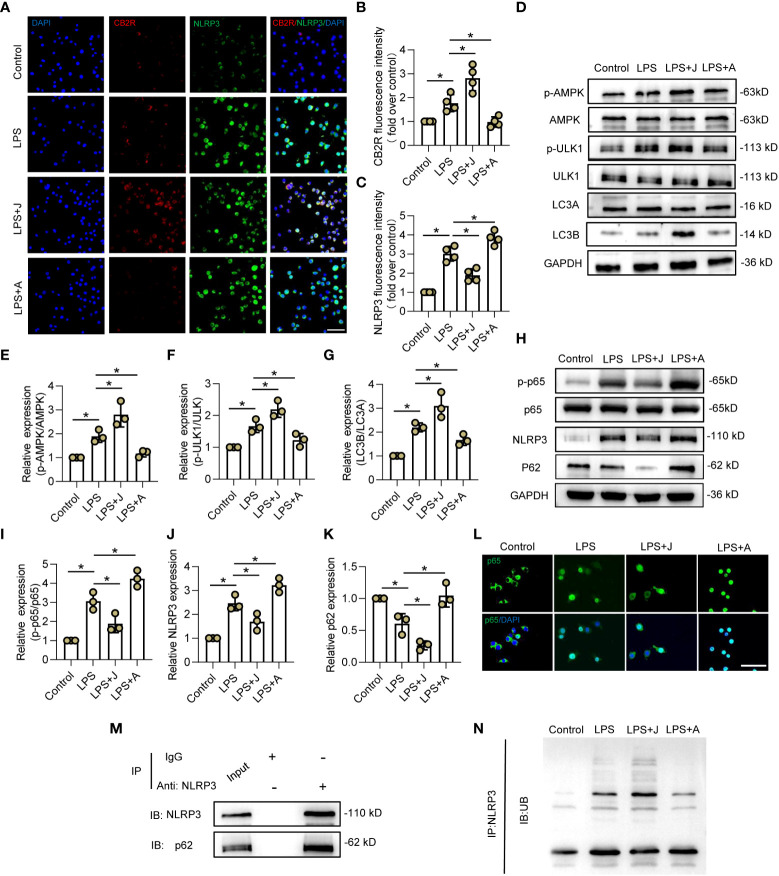
CB2R promoted ubiquitination and autophagy-induced degradation of NLRP3 *via* the AMPK/ULK signaling pathway. **(A)** IF staining of CB2R (red) and NLRP3 (green) after LPS stimuli for 24 h with CB2R activation by JWH-133 **(J)** or inactivation by AM630 **(A)**. Scale bar = 50 μm. **(B)** Quantitative analysis of CB2R. **(C)** Quantitative analysis of NLRP3. **(D)** Representative protein bands of p-AMPK, AMPK, p-ULK1, ULK1, LC3A, and LC3B in each group after 24 h of LPS insult. **(E)** Quantitative analysis of the p-AMPK/AMPK ratio. **(F)** Quantitative analysis of the p-ULK1/ULK1 ratio. **(G)** Quantitative analysis of the LC3B/LC3A ratio. **(H)** Representative protein bands of p-p65, p65, NLRP3, and p62 after 24 h of LPS stimulation. **(I)** Quantitative analysis of the p-p65/p65 ratio. **(J)** Quantitative analysis of NLRP3. **(K)** Quantitative analysis of p62. **(L)** IF staining of CB2R (red) and NLRP3 (green) after LPS stimulation for 24 h with CB2R activation by JWH-133 or CB2R inactivation by AM630. Scale bar = 50 μm. **(M)** Representative protein bands of NLRP3 and p62 in BV2 microglia after IP assay using an Ab against NLRP3. **(N)** Representative protein bands of ubiquitin linkage-specific K48 bound to NLRP3. ^*^ *p* < 0.05.

To elucidate the specific mechanism underlying CB2R-regulated polarization of microglia, the relationship between NLRP3 and CB2R was determined by IF staining. As shown in **Figure 3A**, stimulation with LPS increased expression of CB2R and NLRP3, while treatment with JWH-133 increased expression of CB2R, but decreased that of NLRP3, and treatment with AM630 had opposite effects (**Figures 3B, C**). Furthermore, the expression levels of the autophagy-related proteins adenosine monophosphate-activated protein kinase (AMPK), unc-51-like autophagy activating kinase 1 (ULK1), and microtubule-associated proteins 1A/1B light chain 3 (LC3A/B) were determined by WB analysis. The results showed that treatment with JWH-133 promoted phosphorylation of AMPK and ULK-1, leading to significantly increased expression of LC3B, which was reversed following CB2R inhibition by AM630 (**Figures 3D–G**). Moreover, activation of CB2R by JWH-133 decreased LPS-induced upregulation of NLRP3 and significantly reduced expression of p62, while treatment with AM630 had opposite effects (**Figures 3H, J, K**). Moreover, activation of CB2R inhibited phosphorylation of p65, while inhibition of CB2R fueled phosphorylation and nuclear expression of p65 (**Figures 3H, I, L**). The results of IP analysis showed that p62 was pulled down by NLRP3, indicating a direct interaction between NLRP3 and p62 (**Figure 3M**). Furthermore, in LPS-stimulated HEK-293T cells, treatment with JWH-133 increased binding of ubiquitin linkage-specific K48 to NLRP3, while treatment with AM630 had opposite effects (**Figure 3N**). These findings indicated that CB2R might also regulate ubiquitination of NLRP3 in LPS-stimulated microglia.

### CB2R alleviated inflammation by promoting NLRP3-autophagosome formation in injured cords post-SCI

**Figure 4:**
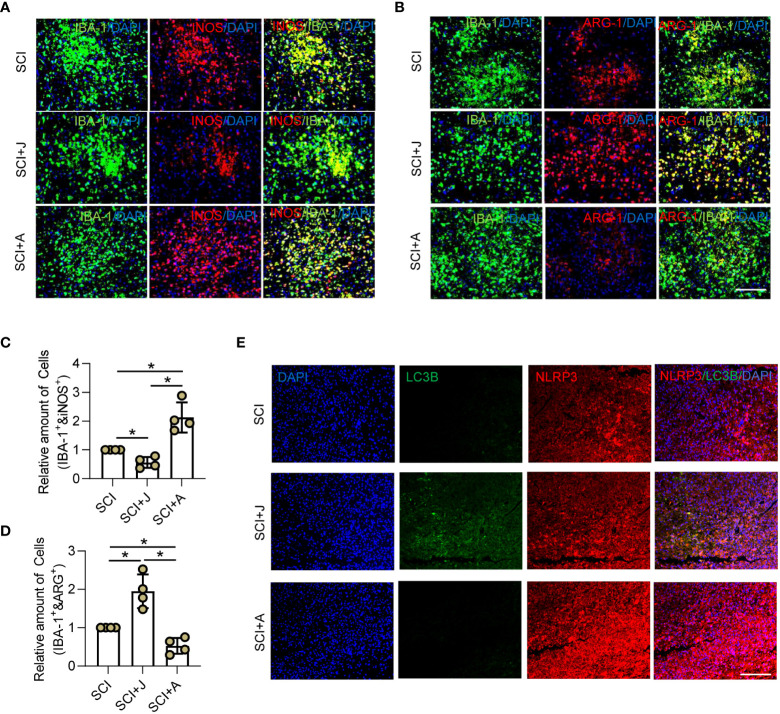
CB2R alleviated inflammation by promoting NLRP3-autophagosome formation in injured cords post-SCI. On day 3 post-SCI with CB2R activation by JWH-133 or inactivation by AM630, **(A)** IF staining of iNOS (red) and IBA-1 (green); **(B)** IF staining of ARG-1 (red) and IBA-1 (green). Scale bar = 100 μm. **(C)** Quantitative analysis of the number of iNOS^+^ microglia. **(D)** Quantitative analysis of the number of ARG-1^+^ microglia. **(E)** IF staining of NLRP3 (red) and LC3B (green) on day 3 post-SCI. Scale bar = 100 μm. ^*^ *p* < 0.05.

*In vivo*, the microglial polarization markers iNOS and ARG-1 with IBA-1 were measured. IF staining showed that the proportion of iNOS+ microglia was increased, while that of ARG-1+ microglia was decreased post-SCI. However, administration of JWH-133 markedly reduced the proportion of M1 microglia and increased that of the M2 subtype, while treatment with AM630 had opposite effects (**Figures 4A–D**). The WB results showed that JWH-133 treatment significantly increased phosphorylation of APMK and ULK1 as well as the LC3B/LC3A ratio, but markedly decreased NLRP3 expression, while treatment with AM630 had opposite effects (**Supplementary Figures 2A–E**). In addition, increased NLRP3 expression was mitigated by JWH-133-induced increased LC3B expression on day 3 post-SCI, while treatment with AM630 increased expression of NLRP3 and LC3B (**Figure 4E**). These results suggested that activated CB2R might protect against neuroinflammation by promoting the formation of the NLRP3-autophagosome.

### CB2R ameliorated secondary neuronal apoptosis and demyelination post-SCI

**Figure 5:**
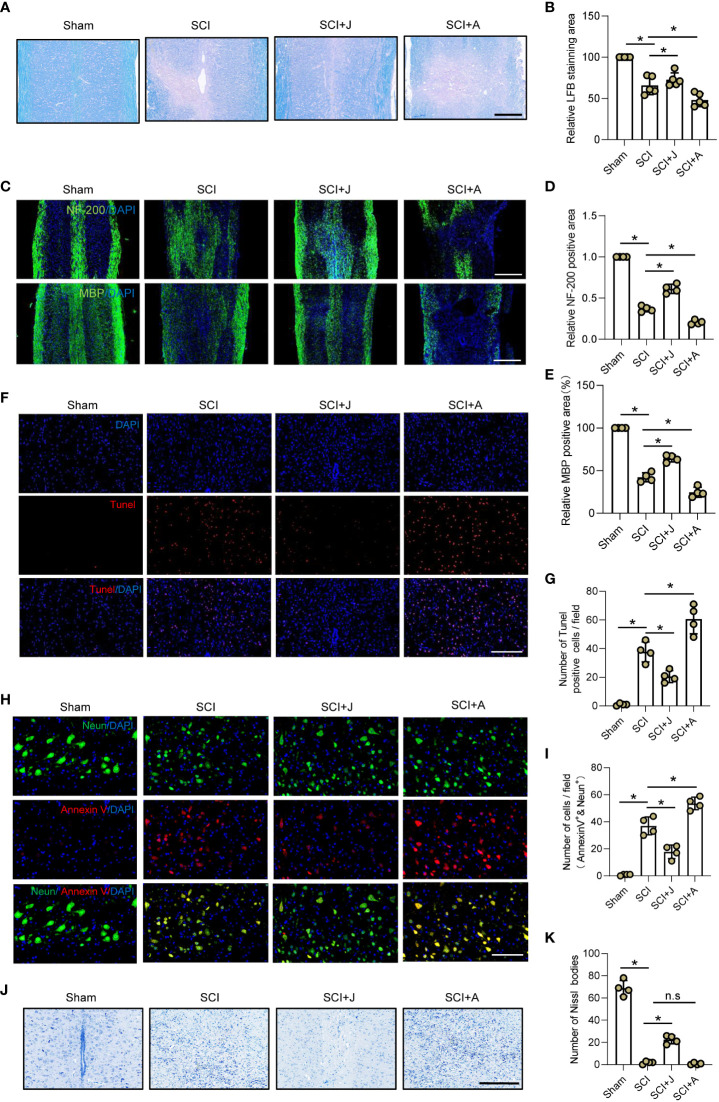
CB2R ameliorated secondary neuronal apoptosis and demyelination post-SCI. **(A)** LFB staining of spinal cords on day 7 post-SCI. Scale bar = 200 μm. **(B)** Quantitative analysis of LFB-labeled area. **(C)** IF staining of NF200 or myelin basic protein (green) on day 7 post-SCI. Scale bar = 500 μm. **(D)** Quantitative analysis of NF200. **(E)** Quantitative analysis of myelin basic protein. **(F)** TUNEL staining of spinal cords on day 7 post-SCI. Scale bar = 100 μm. **(G)** Quantitative analysis of TUNEL-positive cells. **(H)** IF staining of Neun (green) and Annexin V (red) on day 7 post-SCI. Scale bar = 100 μm. **(I)** Quantitative analysis of Annexin V-positive neurons. **(J)** Nissl staining of spinal cords on day 7 post-SCI. Scale bar = 200 μm. **(K)** Quantitative analysis of the number of neurons. ^*^ *p* < 0.05. n.s means no significance.

Given that CB2R was found to play a protective role in secondary injury, the LFB staining profiles of the injured spinal cord on day 7 post-SCI were compared among the groups. The results showed that treatment with JWH-133 resulted in a larger area of myelin as compared to the SCI mice, while treatment with AM630 caused significant myelin loss (**Figures 5A, B**), which was consistent with IF staining of myelin basic protein (**Figures 5C, E**). IF staining showed that treatment with JWH-133 increased the amount of neurofilaments, while treatment with AM630 further aggravated loss of neurofilaments as compared to the SCI mice (**Figures 5C, D**). TUNEL staining revealed that treatment with JWH-133 significantly reduced the amount of dead cells, while treatment with AM630 significantly increased cell death, as compared to the SCI mice (**Figures 5F, G**). Moreover, the number of apoptotic neurons was determined by staining with Annexin V, a marker of early apoptosis, IF analysis, and Nissl staining. The results showed that, as compared to the SCI mice, administration of JWH-133 resulted in fewer apoptotic neurons and a larger number of viable neurons, while treatment with AM630 produced opposite effects (**Figures 5H–K**).

### CB2R mitigated expansion of neuronal damage and formation of glial scarring post-SCI

**Figure 6:**
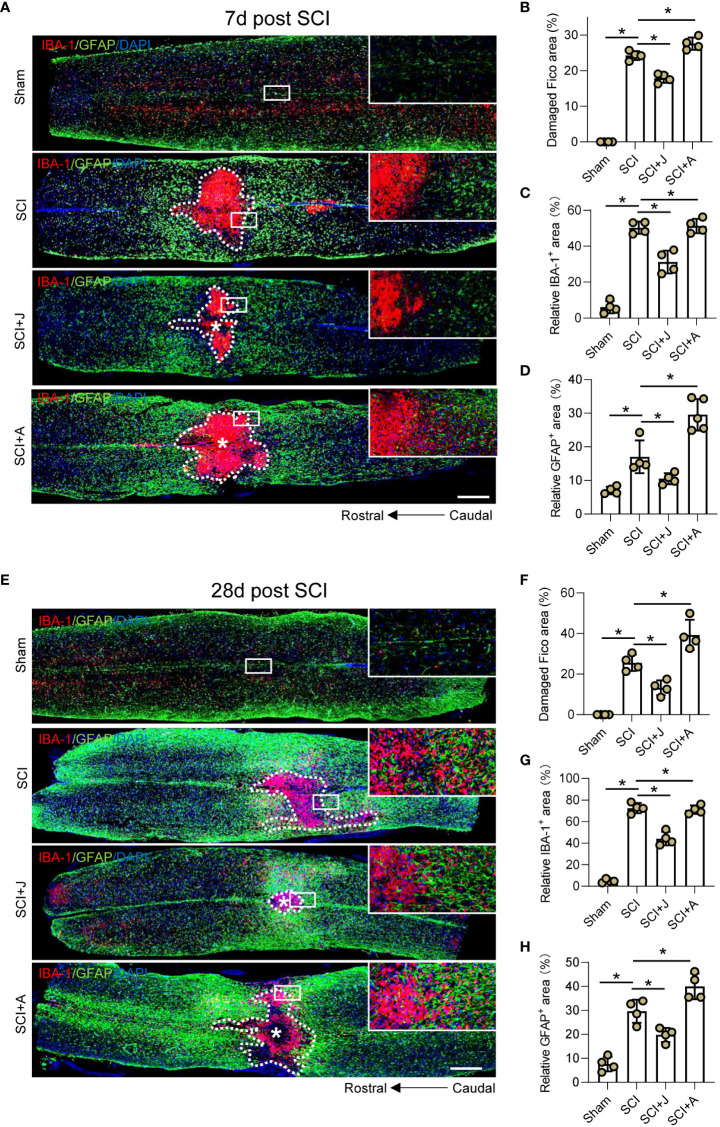
CB2R mitigated expansion of neuronal damage and formation of glial scarring post-SCI. **(A)** IF staining of GFAP (green) and IBA-1 (red) on day 7 post-SCI. Scale bar = 500 μm. Quantitative analysis of the area of injured foci inside the white dotted line **(B)**, microglia-occupied area, **(C)** and astrocyte-occupied area **(D)** on day 7 post-SCI. **(E)** IF staining of GFAP (green) and IBA-1 (red) on day 28 post-SCI. Scale bar = 500 μm. Quantitative analysis of the area of injured foci inside the white dotted line **(F)**, microglia-occupied area, **(G)** and astrocyte-occupied area **(H)** on day 28 post-SCI. ^*^ *p* < 0.05.

Since glial scarring was initiated on day 7 and completed on day 28 post-SCI, IF staining of the injured foci on days 7 and 28 post-SCI was compared. IF staining on day 7 post-SCI showed that JWH-133 administration significantly reduced the extent of neuronal damage in areas occupied by microglia and astrocytes, as compared to the SCI mice, while administration of AM630 had opposite effects (**Figures 6A–D**). Assessment of these indices showed consistent trends on days 7 and 28 post-SCI during the period of spontaneous healing (**Figures 6E–H**).

### CB2R attenuated histological and functional damage post-SCI

**Figure 7:**
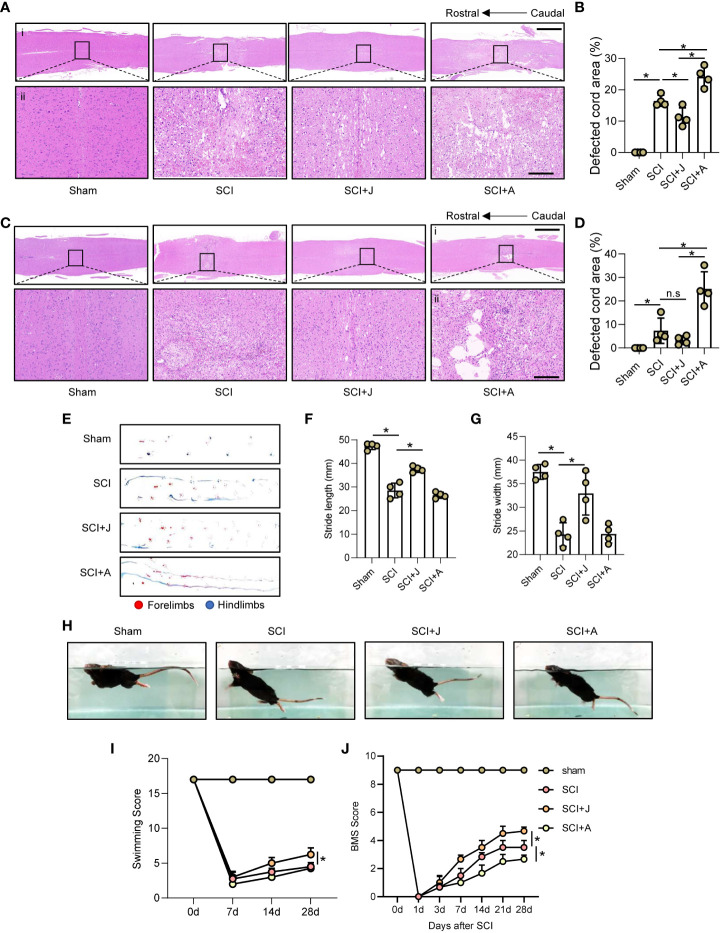
CB2R attenuated histological and functional damage post-SCI. **(A)** Representative images of H&E staining of spinal cords on day 3 post-SCI (injured cords in black frames). Scale bar **(i)** = 600 μm **(ii)** = 100 μm. **(B)** Quantitative analysis of defected area by H&E staining. **(C)** H&E staining of spinal cords on day 3 post-SCI (injured cords in black frames). Scale bar: **(i)** = 600 μm, **(ii)** = 100 μm. **(D)** Quantitative analysis of defected area by H&E staining. **(E)** Mouse footprint assay on day 28 post-SCI. Quantitative analysis of stride length **(F)** and width **(G)** of the hindlimbs of mice post-SCI. **(H)** Representative images of a mouse swimming on day 28 post-SCI. Statistical analysis of the Louisville Swim Scale score **(I)** and Basso Mouse Scale **(J)** over a 28-day period. ^*^ *p* < 0.05. n.s means no significance.

H&E staining showed that as compared to the SCI group on day 3 post-SCI, administration of JWH-133 reduced the areas of hemorrhage and damage of the injured cord tissues, while administration of AM630 increased the areas of hemorrhage and significantly increased the damaged areas (**Figures 7A, B**; **Supplementary Figure 1A**). Furthermore, H&E staining on day 28 post-SCI showed that treatment with JWH-133 prevented further histological damage to the injured foci, while treatment with AM630 exacerbated tissue damage, as compared to the SCI mice (**Figures 7C, D**; **Supplementary Figure 1B**). The results of the footprint assay showed that treatment with JWH-133 lowered the frequency of dorsiflexion of the hindlimbs with longer stride length and width, while treatment with AM630 was associated with lower crawling indices, as compared to the SCI mice (**Figures 7E–G**). The results of the swimming test showed that JWH-133-treated mice exhibited a smaller angle ranging from the trunk to the water surface with a higher frequency of hindlimb movements, while AM630-treated mice seldom exhibited any evident difference, as compared to the SCI mice on day 28 post-SCI (**Figure 7H**). Therefore, the Louisville Swim Scale scores of the JWH-133-treated mice were higher than those of the SCI mice and AM630-treated mice beginning on day 14 post-SCI (**Figure 7I**). The Basso Mouse Scale scores also showed that, as compared to the SCI mice, treatment with JWH-133 significantly improved locomotion, while treatment with AM630 significantly decreased locomotion beginning on day 7 post-SCI (**Figure 7J**).

## Discussion

The findings of the present study suggested that the neuroprotective effects of CB2R activation in SCI were partly regulated by two main mechanisms: (i) attenuation of neuroinflammatory responses and reversed microglial polarization through inactivation of NLRP3 *via* AMPK/ULK1 signaling-induced autophagy and (ii) CB2R mitigation of neuroinflammation-mediated secondary neuropathology due to myelin loss, neuron apoptosis, and glial scar accumulation post-SCI.

LPS provokes inflammatory responses in multiple immune cells, including macrophages, T cells, and microglia (33, 34). Hence, LPS is often used to establish *in vitro* aseptic inflammatory models to mimic the pathogenesis of various neuro-immune diseases, such as autoimmune encephalomyelitis, multiple sclerosis, and depression (35–37). Of note, LPS is also widely used to induce microglial inflammation in mimetic models of SCI (38–40). LPS binds to Toll-like receptor 4 and promotes activation of p65, which then translocates to the nucleus and triggers the transcription of pro-inflammatory molecules (41, 42). The results of the current study confirmed that CB2R expression was significantly increased both in the cord tissues of SCI mice and in LPS-stimulated microglia, which then inhibited activation of p65, leading to decreased translocation to the nucleus. A previous report also suggested that CB2R activation could mitigate LPS-induced neuroinflammation (43), but the underlying molecular mechanism regulated by CB2R has not yet been elucidated. The results of the current study showed that pharmacological activation of CB2R by JWH-133 inhibited LPS-induced proinflammatory transition of the M1 subtype and increased the proportion of immunoregulatory M2 microglia *in vitro*, which was reversed by treatment with the CB2R antagonist AM630. These findings indicated that inhibition of neuroinflammation *via* CB2R potentially occurred through unidentified signaling pathways related to immunoregulation.

NLRP3, a classic biomarker of inflammation, responds to trauma *via* regulating activation of caspase-1 and maturation of IL-1β, thereby promoting inflammation-dominated neuropathology post-SCI (44, 45). Accumulating evidence indicates that either suppression of NLRP3 expression or promotion of cell autophagy ameliorates neuroinflammation-induced neurological injury, although the underlying mechanisms remain unclear. Numerous mechanisms have been proposed to explain the interactions between the NLRP3 inflammasome and autophagy, including autophagy activation by inflammatory signaling *via* limited IL-1β production and targeting ubiquitinated inflammasomes (13). In neurological diseases, non-coding RNA, bio-enzymes, and small molecular compounds have been implicated in the interplay between ubiquitination and autophagy of NLRP3 (46–48). In LPS-stimulated microglia, CB2R activation decreased NLRP3 expression associated with formation of autophagosomes, resulting in accumulation of LC3B and exhaustion of p62. Consistently, Shao et al. (49) demonstrated that alleviation of experimental autoimmune encephalomyelitis by CB2R activation was linked to activation of autophagosomes and inhibition of the NLRP3 inflammasome. In contrast, the results of the present study suggest that CB2R regulates NLRP3 ubiquitination by ubiquitin linkage-specific K48 and promotes binding of NLRP3 with p62, an autophagy substrate, and LC3B, an autophagy activator. K48-linked polyubiquitination of NLRP3 mediates self-degradation and reduces cellular NLRP3 levels, which are essential for inactivation of the NLRP3 inflammasome (50) and p62-dependent selective autophagy also inhibited the capacity of NLRP3 inflammasome-related proteins to attenuate inflammation-related pathology (51, 52). These findings confirmed that CB2R activation indeed mitigated neuroinflammation targeting polarization of microglia by regulating autophagy- and ubiquitination-mediated degradation of the NLRP3 inflammasome. AMPK, a nutrient and energy sensor that controls mitochondrial homeostasis, is activated by multiple physio-pathological insults, resulting in inflammation (53, 54), and subsequent phosphorylation of ULK1, resulting in activation of autophagosomes (55). Strikingly, CB2R has been implicated in upregulation of AMPK expression in models of aging, inflammation, and hyperglycemia (55–57), implying that CB2R might also participate in the regulation of autophagy. Interestingly, the results of the present study showed that activation of CB2R increased AMPK/ULK1 phosphorylation, leading to increased expression of LC3B, indicating that CB2R activation in a state of neuroinflammation facilitated autophagy *via* activation of the AMPK/ULK1 axis.

An increase in the proportion of pro-inflammatory M1 microglia that express iNOS with a decrease in the proportion of anti-inflammatory M2 microglia expressing ARG-1 in the early stage of SCI plays a crucial role in neuroinflammation (58, 59). Therefore, upregulation of iNOS and downregulation of ARG-1 promote deteriorative secondary neuropathy and more intense neuroinflammation in the injured cord tissues post-SCI. The results of the *in vivo* experiments found that pro-inflammatory M1 microglia predominantly expressing iNOS and penuriously expressing ARG-1 had accumulated in the vicinity of the injured cord, while CB2R activation *via* administration of JWH-133 resulted in increased expression of ARG-1 with reduced levels of iNOS in microglia around the injury. In contrast, treatment with the CB2R antagonist AM630 promoted the formation of M1, rather than M2, microglia, suggesting that CB2R activation limited inflammation by manipulating polarization of microglia in the injured spinal cord. Accordingly, CB2R activation reduced secondary pathology like neuron loss, demyelination, and glial scarring, thereby improving functional recovery. However, these corrections were neutralized in AM630-treated mice, suggesting that CB2R may play a potent neuroprotective role in addition to anti-inflammatory activities. In summary, CB2R attenuated inflammatory responses *via* targeting microglial differentiation by facilitating autophagy- and ubiquitination-induced degradation of the NLRP3 inflammasome. CB2R expression in SCI mice reduced the area of glial scarring, rescued the deficiency of neurons and myelin, and improved recovery of locomotor function, in part, by inhibiting neuroinflammation.

These preliminary findings verified the role of CB2R in post-SCI neuroinflammation and subsequent neuropathology. Hence, further research is warranted to explore therapeutic strategies targeting CB2R and other functions of CB2R in neurons to reveal their potential immunotherapeutic value for SCI.

## Data availability statement

The original contributions presented in the study are included in the article/**Supplementary Material**. Further inquiries can be directed to the corresponding authors.

## Ethics statement

This study was reviewed and approved by the Institutional Animal Care and Use Committee of Nanjing Medical University.

## Author contributions

FJ and ZZ conceived and designed the experiments. FJ performed the cell experiments. MX and YZ performed the animal experiments. JCh and JCa analyzed the data. ZQ wrote the paper. LY funded and supervised the research. All authors contributed to the article and approved the submitted version.

## Funding

This study was supproted by 2022 Lifting Project for Young Scientific and Technological Talents Funded by Jiangsu Association of Science and Technology (To LY Grant No#:TJ-2022-033).

## Acknowledgments

We thank the Key Laboratory of Taizhou People’s Hospital for technological support and the use of equipment.

## Conflict of interest

The authors declare that the research was conducted in the absence of any commercial or financial relationships that could be construed as a potential conflict of interest.

## Publisher’s note

All claims expressed in this article are solely those of the authors and do not necessarily represent those of their affiliated organizations, or those of the publisher, the editors and the reviewers. Any product that may be evaluated in this article, or claim that may be made by its manufacturer, is not guaranteed or endorsed by the publisher.

## Supplementary material

Supplementary Figure 1 Photos of 4% paraformaldehyde-fixed spinal cords on days 3 (**A**) and 28 (**B**) post-SCI in each group.

Supplementary Figure 2 (**A**) Representative protein bands including of p-AMPK, AMPK, p-ULK1, ULK1, LC3A, LC3B, and NLPR3 in each group 3 days after SCI. (**B**) Quantitative analysis of ratio of the p-AMPK/AMPK ratio. (**C**) Quantitative analysis of the p-ULK1/ULK1 ratio. (**D**) Quantitative analysis of the LC3B/LC3A ratio. (**E**) Quantitative analysis of NLRP3 expression.
