# Supplementary figures and images for: Cannabinoid receptor-2 attenuates neuroinflammation by promoting autophagy-mediated degradation of the NLRP3 inflammasome post spinal cord injury
Source: Front Immunol. 2022 Sep 26;13:993168. doi: 10.3389/fimmu.2022.993168 (PMC9553321; doi:10.3389/fimmu.2022.993168)

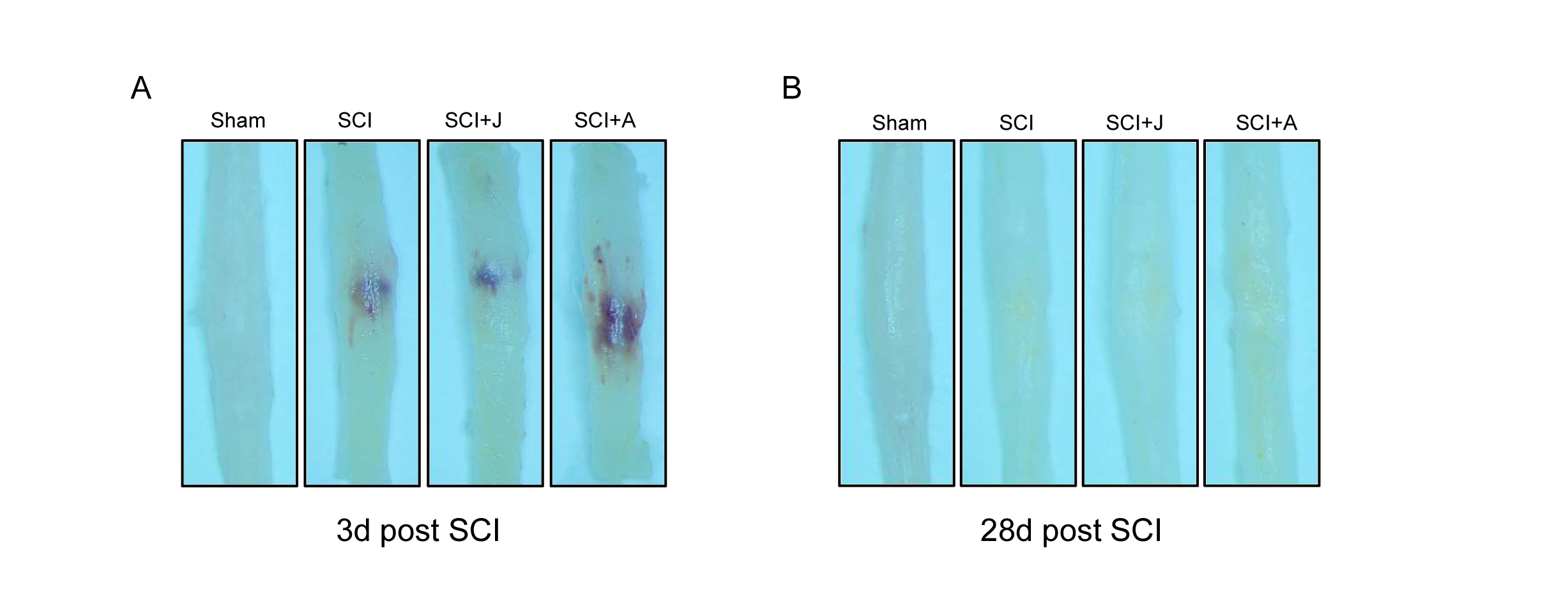

Supplement: Supplementary Figure 1 — Photos of 4% paraformaldehyde-fixed spinal cords on days 3 (A) and 28 (B) post-SCI in each group. [file Image_1.tif]

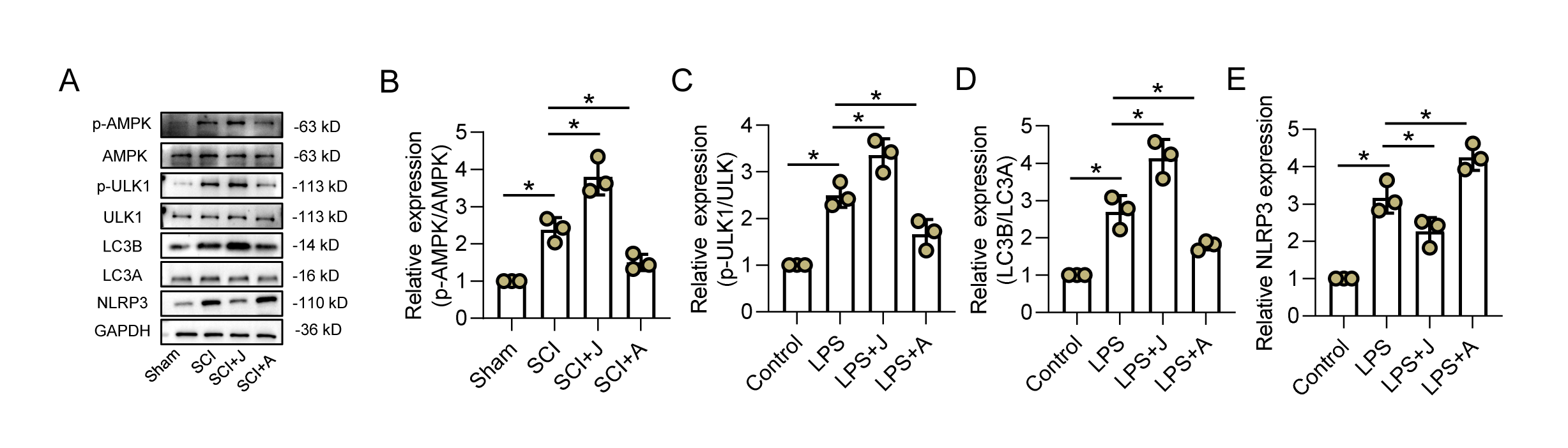

Supplement: Supplementary Figure 2 — (A) Representative protein bands including of p-AMPK, AMPK, p-ULK1, ULK1, LC3A, LC3B, and NLPR3 in each group 3 days after SCI. (B) Quantitative analysis of ratio of the p-AMPK/AMPK ratio. (C) Quantitative analysis of the p-ULK1/ULK1 ratio. (D) Quantitative analysis of the LC3B/LC3A ratio. (E) Quantitative analysis of NLRP3 expression. [file Image_2.tif]
